# 4,6-Di-*tert*-butyl-2,3-di­hydroxy­benzalde­hyde

**DOI:** 10.1107/S1600536813025488

**Published:** 2013-09-18

**Authors:** Max Arsenyev, Eugene Baranov, Sergey Chesnokov, Gleb Abakumov

**Affiliations:** a Laboratory of Free Radical Polymerization, G.A. Razuvaev Institute of ­Organometallic Chemistry of the Russian Academy of Science, Tropinina str, 49, Nizhny Novgorod, 603950, Russian Federation; b Group of X-Ray Diffraction Investigations, G.A. Razuvaev Institute of Organometallic Chemistry of the Russian Academy of Science, Tropinina str, 49, Nizhny Novgorod, 603950, Russian Federation; c Laboratory of the Chemistry of Organometallic Compounds, G.A. Razuvaev Institute of Organometallic Chemistry of the Russian Academy of Science, Tropinina str, 49, Nizhny Novgorod, 603950, Russian Federation

## Abstract

The title compound, C_15_H_22_O_3_, crystallizes with two independent mol­ecules in the asymmetric unit. In each mol­ecule, one hy­droxy group (at position 2) is involved in an intra­molecular O—H⋯O hydrogen bond, and another one (at position 3) exhibits bifurcated hydrogen-bonding being involved in intra- and inter­molecular O—H⋯O inter­actions. In the crystal, O—H⋯O hydrogen bonds link alternating independent mol­ecules into chains running along [010].

## Related literature

For the crystal structure of 2,3-di­hydroxy­benzaldehyde, see: Ng (2005 ▶). For applications of Shiff base ligands based on 2,3-di­hydroxy­benzaldehyde, see: Albrecht *et al.* (2004 ▶); Furutachi *et al.* (2010 ▶); Belmonte *et al.* (2012 ▶).
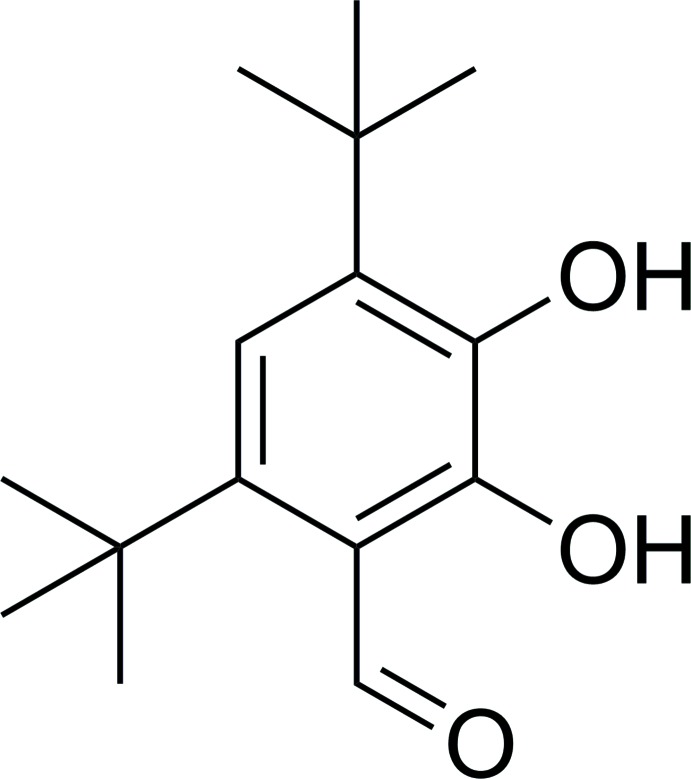


## Experimental

### 

#### Crystal data

C_15_H_22_O_3_
*M* *_r_* = 250.33
Triclinic, 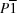

*a* = 9.3113 (9) Å
*b* = 10.6511 (10) Å
*c* = 15.3962 (15) Å
α = 95.242 (2)°
β = 103.085 (2)°
γ = 95.492 (2)°
*V* = 1470.4 (2) Å^3^
*Z* = 4
Mo *K*α radiation
μ = 0.08 mm^−1^
*T* = 100 K
0.70 × 0.16 × 0.16 mm

#### Data collection

Bruker SMART APEX CCD area-detector diffractometer
Absorption correction: multi-scan (*SADABS*; Sheldrick, 2008 ▶) *T*_min_ = 0.948, *T*_max_ = 0.988
8903 measured reflections
5740 independent reflections
4411 reflections with *I* > 2σ(*I*)
*R*_int_ = 0.018

#### Refinement

*R*[*F* ^2^ > 2σ(*F* ^2^)] = 0.046
*wR*(*F* ^2^) = 0.130
*S* = 1.06
5740 reflections
361 parameters
H atoms treated by a mixture of independent and constrained refinement
Δρ_max_ = 0.25 e Å^−3^
Δρ_min_ = −0.21 e Å^−3^

### 

Data collection: *SMART* (Bruker, 2008 ▶); cell refinement: *SAINT* (Bruker, 2008 ▶); data reduction: *SAINT*; program(s) used to solve structure: *SHELXTL* (Sheldrick, 2008 ▶); program(s) used to refine structure: *SHELXTL*; molecular graphics: *SHELXTL*; software used to prepare material for publication: *SHELXTL*.

## Supplementary Material

Crystal structure: contains datablock(s) global, I. DOI: 10.1107/S1600536813025488/cv5427sup1.cif

Structure factors: contains datablock(s) I. DOI: 10.1107/S1600536813025488/cv5427Isup2.hkl

Supplementary material file. DOI: 10.1107/S1600536813025488/cv5427Isup3.cml

Additional supplementary materials:  crystallographic information; 3D view; checkCIF report

## Figures and Tables

**Table 1:** Hydrogen-bond geometry (Å, °)

*D*—H⋯*A*	*D*—H	H⋯*A*	*D*⋯*A*	*D*—H⋯*A*
O1*A*—H1*A*⋯O2*A*	0.885 (15)	2.169 (15)	2.6360 (10)	112.4 (11)
O2*A*—H2*A*⋯O3*A*	1.154 (14)	1.484 (14)	2.5013 (10)	142.7 (12)
O1*B*—H1*B*⋯O2*B*	0.883 (17)	2.212 (16)	2.6443 (10)	109.7 (12)
O2*B*—H2*B*⋯O3*B*	0.974 (17)	1.608 (16)	2.5046 (10)	150.9 (16)
O1*B*—H1*B*⋯O3*A*	0.883 (17)	1.916 (17)	2.7485 (11)	156.4 (15)
O1*A*—H1*A*⋯O3*B* ^i^	0.885 (15)	1.912 (15)	2.7289 (11)	152.9 (13)

Symmetry code: (i) 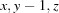.

**Fig. 1.**
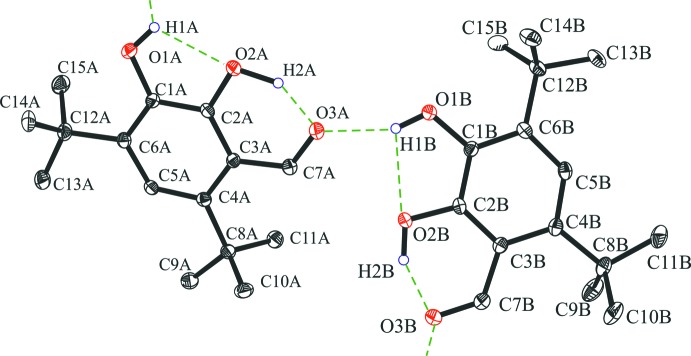
Two independent molecules of the title compound showing the atomic numering and 30% probability displacement ellipsoids. Dashed lines denote hydrogen bonds. C-bound H atoms omitted for clarity.

**Fig. 2.**
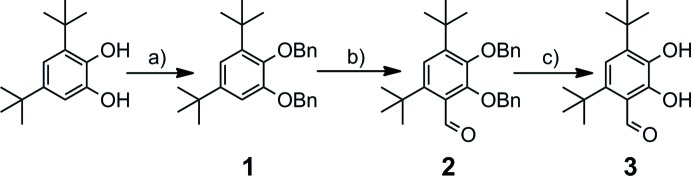
Synthesis of sterically hindered salicylic aldehyde 3. Reagents and conditions: a) benzyl chloride (BnCl), K2CO3, DMF (98%); b) butyllithium (BuLi) in hexane, tetramethylethylenediamine (TMEDA); DMF; H2O (71%); c) BCl3 in CH2Cl2; H2O (85%).

## supplementary crystallographic information

### 1. Comment

2,3-Dihydroxybenzaldehyde is well known building-block for preparing Schiff-base ligands containining catechol fragment. These ligands are used for the synthesis of polynuclear metal complexes (Belmonte *et al.*, 2012), supramolecular compounds (Albrecht *et al.*, 2004) and catalysts (Furutachi *et al.*, 2010). Here we report the crystal structure of the title compound (I).

The asymmetric unit of (I) contains two independent molecules (Fig. 1) with non-typical arrangement of *tert*-butyl groups (*orto*- and *para*- despite *meta*-position relative to CHO-group). All bond lengths and angles in (I) are normal and correspond to those observed in the related 2,3-dihydroxybenzaldehyde (Ng, 2005). The structure shows two types of O—H···O hydrogen bonds (Table 1) - intra- and intermolecular ones.

In the crystal, intermolecular O—H···O hydrogen bonds link alternating independent molecules into chains running in [010] (Fig. 1).

### 2. Experimental

4,6-Di-*tert*-butyl-2,3-dihydroxybenzaldehyde was synthesized by following scheme (Fig. 2).

1,2-Bis(benzyloxy)-3,5-di-*tert*-butylbenzene (1). Mixture of 3,5-di-*tert*-butyl-catechol (22.2 g, 0.1 mol), benzyl chloride (23.0 ml, 0.2 mol) and K_2_CO_3_ (27.6 g, 0.2 mol) in DMF (100 ml) was heated at 90°C for 24 h under argon atmosphere. After cooling, water (300 ml) was added to reaction mixture and the product was extracted by hexane (3*200 ml). Extract was dried by Na_2_SO_4_. The solvent was evaporated and the product was dried under vacuum. The yield was 39.4 g (98%). m.p.=86-87°C dH (200 MHz CDCl_3_) 7.48-7.28 (m, 10H, 2Ph), 7.02 (d, 1H, C_ar_-H, J_HH_=2.2 Hz), 6.95 (d, 1H, C_ar_-H, J_HH_=2.2 Hz), 5.16 (s, 2H, CH_2_Ph), 5.11 (s, 2H, CH_2_Ph), 1.43 and 1.32 (s, both 9H, t-Bu). dC (50 MHz CDCl_3_) 151.85, 145.71, 145.46, 142.73, 138.57, 137.33, 128.45, 128.22, 127.83, 127.79, 127.58, 127.33, 116.55, 110.78, 73.50, 71.44, 35.48, 34.80, 31.56, 30.87.

2,3-bis(benzyloxy)-4,6-di-*tert*-butylbenzaldehyde (2). The compound 1 (20.1 g, 0.05 mol) was dissolved in THF (200 ml), and the solution was cooled to -78°C. TMEDA (7.5 ml, 0.05 mol) and BuLi in hexane (160 ml 0.6 M, 0.1 mol) were added to the mixture. It was stirred for 3 h and DMF (7.7 ml, 0.1 mol) was added. Mixture was stirred at -78°C for 3 h and then was warmed to room temperature, and stirring was continued overnight. The resulting mixture was diluted by water (300 ml) and neutralized by conc. HCl. The mixture of 1 and 2 was extracted by hexane (3*200 ml). The solvent was evaporated and the crude product was purified by column chromatography on silica gel (eluent hexane:ethyl acetate 40:1, second fraction). The yield was 15.3 g (71%). m.p.=84-85°C. dH (200 MHz CDCl_3_) 10.53 (s, 1H, CHO), 7.51-7.31 (m, 11H, 2Ph and C_ar_-H), 5.21 (s, 2H, CH_2_Ph), 4.98 (s, 2H, CH_2_Ph), 1.45 and 1.36 (s, both 9H, t-Bu). dC (50 MHz CDCl_3_) 196.65, 151.76, 149.07, 146.09, 144.31, 137.89, 136.49, 132.26, 128.88, 128.41, 128.36,128.22, 127.56, 127.16, 120.83, 75.85, 73.69, 36.13, 35.86, 32.02, 30.53.

4,6-Di-*tert*-butyl-2,3-dihydroxybenzaldehyde (3). 0.1 M solution of BCl_3_ in CH_2_Cl_2_ (60 ml) was added with cooling (0°C) to 2 (12.9 g, 0.03 mol) in CH_2_Cl_2_ (30 ml). Reaction mixture was stirred for 24 h. Water (50 ml) was added to the mixture, and stirring was continued for 24 h. Product was extracted by CH_2_Cl_2_ and washed by water (4*50 ml). Extract was dried by Na_2_SO_4_ and the solvent was evaporated. 3 was recrystallized from methanol (50 ml). Yellow crystalline (6.4 g, 85%). m.p.=115-116°C. nmax (nujol) 3620.86 (nar.), 3529-3240 (br.), 1624 (C=O)cm-1. dH (200 MHz CDCl_3_) 12.92 (s, 1H, OH), 10.71 (s, 1H, CHO), 6.88 (s, 1H, C_ar_-H), 5.99 (s, 1H, OH), 1.42 and 1.49 (s, both 9H, t-Bu). dC (50 MHz CDCl_3_) 196.58, 151.93, 143.24, 141.77, 141.73, 115.99 (C-H), 115.19, 35.75, 35.72, 33.76, 29.00. Anal. Calcd for C15H22O3: C 71.97, H 8.86. Found: C 71.89, H 8.90.

### 3. Refinement

C-bound H atoms, excluding H7A and H7B, were placed in calculated positions and were refined in the riding model. The rest H atoms were located on a difference map and were refined isotropically.

### Crystal data

**Table:**

C_15_H_22_O_3_	*Z* = 4
*M**_r_* = 250.33	*F*(000) = 544
Triclinic, *P*1	*D*_x_ = 1.131 Mg m^−^^3^
Hall symbol: -P 1	Melting point: 115 K
*a* = 9.3113 (9) Å	Mo *K*α radiation, λ = 0.71073 Å
*b* = 10.6511 (10) Å	Cell parameters from 900 reflections
*c* = 15.3962 (15) Å	θ = 2–30°
α = 95.242 (2)°	µ = 0.08 mm^−^^1^
β = 103.085 (2)°	*T* = 100 K
γ = 95.492 (2)°	Prism, yellow
*V* = 1470.4 (2) Å^3^	0.70 × 0.16 × 0.16 mm

### Data collection

**Table:**

Bruker SMART APEX CCD area-detector diffractometer	5740 independent reflections
Radiation source: fine-focus sealed tube	4411 reflections with *I* > 2σ(*I*)
Graphite monochromator	*R*_int_ = 0.018
ω scans	θ_max_ = 26.0°, θ_min_ = 2.2°
Absorption correction: multi-scan (*SADABS*; Sheldrick, 2008)	*h* = −11→11
*T*_min_ = 0.948, *T*_max_ = 0.988	*k* = −11→13
8903 measured reflections	*l* = −18→18

### Refinement

**Table:**

Refinement on *F*^2^	0 restraints
Least-squares matrix: full	H atoms treated by a mixture of independent and constrained refinement
*R*[*F*^2^ > 2σ(*F*^2^)] = 0.046	*w* = 1/[σ^2^(*F*_o_^2^) + (0.0779*P*)^2^ + 0.0742*P*] where *P* = (*F*_o_^2^ + 2*F*_c_^2^)/3
*wR*(*F*^2^) = 0.130	(Δ/σ)_max_ < 0.001
*S* = 1.06	Δρ_max_ = 0.25 e Å^−^^3^
5740 reflections	Δρ_min_ = −0.21 e Å^−^^3^
361 parameters	

### Special details

**Table:**

Geometry. All e.s.d.'s (except the e.s.d. in the dihedral angle between two l.s. planes) are estimated using the full covariance matrix. The cell e.s.d.'s are taken into account individually in the estimation of e.s.d.'s in distances, angles and torsion angles; correlations between e.s.d.'s in cell parameters are only used when they are defined by crystal symmetry. An approximate (isotropic) treatment of cell e.s.d.'s is used for estimating e.s.d.'s involving l.s. planes.
Refinement. Refinement of *F*^2^ against ALL reflections. The weighted *R*-factor *wR* and goodness of fit *S* are based on *F*^2^, conventional *R*-factors *R* are based on *F*, with *F* set to zero for negative *F*^2^. The threshold expression of *F*^2^ > σ(*F*^2^) is used only for calculating *R*-factors(gt) *etc*. and is not relevant to the choice of reflections for refinement. *R*-factors based on *F*^2^ are statistically about twice as large as those based on *F*, and *R*- factors based on ALL data will be even larger.

### Fractional atomic coordinates and isotropic or equivalent isotropic displacement parameters (Å^2^)

**Table:**

	*x*	*y*	*z*	*U*_iso_*/*U*_eq_	
O1A	0.13058 (9)	−0.38259 (6)	0.24551 (5)	0.0316 (2)	
H1A	0.1319 (16)	−0.3993 (13)	0.1884 (10)	0.054 (4)*	
O2A	0.14084 (8)	−0.22900 (6)	0.12158 (5)	0.02752 (18)	
H2A	0.1392 (16)	−0.1505 (13)	0.0745 (10)	0.057 (4)*	
O3A	0.15955 (9)	−0.01066 (7)	0.07434 (5)	0.02887 (19)	
C1A	0.15563 (11)	−0.25515 (9)	0.27164 (7)	0.0226 (2)	
C2A	0.16112 (11)	−0.17213 (9)	0.20646 (7)	0.0216 (2)	
C3A	0.18605 (10)	−0.03905 (9)	0.22842 (7)	0.0214 (2)	
C4A	0.21238 (11)	0.01227 (9)	0.32024 (7)	0.0223 (2)	
C5A	0.20502 (11)	−0.07376 (9)	0.38215 (7)	0.0229 (2)	
H5AA	0.2218	−0.0407	0.4435	0.027*	
C6A	0.17450 (11)	−0.20648 (9)	0.36057 (7)	0.0232 (2)	
C7A	0.18009 (12)	0.03568 (9)	0.15347 (7)	0.0253 (2)	
H7A	0.1889 (13)	0.1306 (11)	0.1631 (8)	0.031 (3)*	
C8A	0.24859 (12)	0.15602 (9)	0.35317 (7)	0.0261 (3)	
C9A	0.28235 (14)	0.18201 (10)	0.45588 (8)	0.0352 (3)	
H9AA	0.1952	0.1507	0.4768	0.053*	
H9AB	0.3066	0.2736	0.4741	0.053*	
H9AC	0.3668	0.1383	0.4823	0.053*	
C10A	0.11678 (13)	0.22788 (10)	0.31648 (8)	0.0337 (3)	
H10A	0.0299	0.1942	0.3368	0.051*	
H10B	0.0940	0.2169	0.2507	0.051*	
H10C	0.1423	0.3184	0.3385	0.051*	
C11A	0.38925 (13)	0.21161 (10)	0.32590 (8)	0.0331 (3)	
H11A	0.3704	0.2048	0.2603	0.050*	
H11B	0.4719	0.1641	0.3493	0.050*	
H11C	0.4146	0.3011	0.3508	0.050*	
C12A	0.15986 (13)	−0.29441 (10)	0.43265 (7)	0.0295 (3)	
C13A	0.17984 (15)	−0.22047 (11)	0.52559 (8)	0.0369 (3)	
H13A	0.1043	−0.1620	0.5233	0.055*	
H13B	0.2789	−0.1721	0.5437	0.055*	
H13C	0.1691	−0.2801	0.5692	0.055*	
C14A	0.00288 (14)	−0.36849 (11)	0.40655 (8)	0.0382 (3)	
H14A	−0.0111	−0.4201	0.3485	0.057*	
H14B	−0.0713	−0.3086	0.4020	0.057*	
H14C	−0.0088	−0.4240	0.4525	0.057*	
C15A	0.27806 (14)	−0.38673 (11)	0.43956 (8)	0.0387 (3)	
H15A	0.2660	−0.4369	0.3812	0.058*	
H15B	0.2664	−0.4437	0.4846	0.058*	
H15C	0.3772	−0.3385	0.4573	0.058*	
O1B	0.19869 (9)	0.08055 (6)	−0.08108 (5)	0.02929 (19)	
H1B	0.1807 (18)	0.0734 (14)	−0.0277 (11)	0.067 (5)*	
O2B	0.14124 (8)	0.25992 (6)	0.03230 (5)	0.02881 (18)	
H2B	0.1334 (19)	0.3373 (15)	0.0690 (11)	0.075 (5)*	
O3B	0.14462 (10)	0.48947 (7)	0.08636 (5)	0.0382 (2)	
C1B	0.22899 (11)	0.20423 (9)	−0.09443 (7)	0.0229 (2)	
C2B	0.19582 (11)	0.30029 (9)	−0.03624 (7)	0.0227 (2)	
C3B	0.22243 (11)	0.42972 (9)	−0.04678 (7)	0.0242 (2)	
C4B	0.28051 (11)	0.46447 (9)	−0.12054 (7)	0.0241 (2)	
C5B	0.31494 (11)	0.36661 (9)	−0.17478 (7)	0.0247 (2)	
H5BA	0.3557	0.3885	−0.2234	0.030*	
C6B	0.29418 (11)	0.23659 (9)	−0.16335 (7)	0.0230 (2)	
C7B	0.19374 (14)	0.51970 (10)	0.02154 (8)	0.0338 (3)	
H7B	0.2122 (16)	0.6105 (13)	0.0160 (10)	0.055 (4)*	
C8B	0.30439 (12)	0.60314 (10)	−0.14183 (8)	0.0302 (3)	
C9B	0.43000 (15)	0.68113 (11)	−0.06932 (10)	0.0469 (4)	
H9BA	0.4426	0.7687	−0.0837	0.070*	
H9BB	0.5226	0.6436	−0.0671	0.070*	
H9BC	0.4049	0.6809	−0.0108	0.070*	
C10B	0.16044 (14)	0.66445 (11)	−0.15304 (9)	0.0418 (3)	
H10D	0.1763	0.7494	−0.1714	0.063*	
H10E	0.1305	0.6712	−0.0959	0.063*	
H10F	0.0821	0.6121	−0.1991	0.063*	
C11B	0.34989 (16)	0.60891 (11)	−0.23132 (9)	0.0459 (4)	
H11D	0.3606	0.6974	−0.2441	0.069*	
H11E	0.2734	0.5587	−0.2796	0.069*	
H11F	0.4447	0.5743	−0.2274	0.069*	
C12B	0.34396 (12)	0.13435 (10)	−0.22352 (7)	0.0279 (3)	
C13B	0.42121 (14)	0.19175 (11)	−0.29129 (8)	0.0379 (3)	
H13D	0.3519	0.2374	−0.3309	0.057*	
H13E	0.4530	0.1237	−0.3272	0.057*	
H13F	0.5081	0.2508	−0.2591	0.057*	
C14B	0.20742 (14)	0.04340 (11)	−0.27618 (8)	0.0378 (3)	
H14D	0.1382	0.0911	−0.3141	0.057*	
H14E	0.1581	0.0036	−0.2340	0.057*	
H14F	0.2390	−0.0225	−0.3140	0.057*	
C15B	0.45488 (13)	0.06066 (11)	−0.16510 (8)	0.0358 (3)	
H15D	0.5414	0.1194	−0.1319	0.054*	
H15E	0.4869	−0.0042	−0.2035	0.054*	
H15F	0.4072	0.0198	−0.1226	0.054*	

### Atomic displacement parameters (Å^2^)

**Table:**

	*U*^11^	*U*^22^	*U*^33^	*U*^12^	*U*^13^	*U*^23^
O1A	0.0487 (5)	0.0195 (3)	0.0268 (4)	−0.0008 (3)	0.0121 (4)	0.0013 (3)
O2A	0.0381 (4)	0.0244 (3)	0.0206 (4)	0.0004 (3)	0.0105 (3)	0.0001 (3)
O3A	0.0371 (4)	0.0276 (3)	0.0233 (4)	0.0024 (3)	0.0099 (3)	0.0047 (3)
C1A	0.0237 (5)	0.0197 (4)	0.0254 (5)	0.0007 (4)	0.0085 (4)	0.0027 (4)
C2A	0.0216 (5)	0.0247 (5)	0.0187 (5)	0.0008 (4)	0.0069 (4)	0.0005 (4)
C3A	0.0190 (5)	0.0214 (4)	0.0257 (5)	0.0020 (4)	0.0087 (4)	0.0043 (4)
C4A	0.0184 (5)	0.0233 (5)	0.0257 (5)	0.0028 (4)	0.0065 (4)	0.0019 (4)
C5A	0.0233 (5)	0.0252 (5)	0.0205 (5)	0.0017 (4)	0.0072 (4)	0.0006 (4)
C6A	0.0209 (5)	0.0254 (5)	0.0240 (5)	0.0018 (4)	0.0063 (4)	0.0043 (4)
C7A	0.0260 (5)	0.0254 (5)	0.0259 (5)	0.0023 (4)	0.0090 (4)	0.0037 (4)
C8A	0.0289 (5)	0.0225 (5)	0.0268 (5)	−0.0012 (4)	0.0099 (4)	−0.0006 (4)
C9A	0.0444 (7)	0.0274 (5)	0.0314 (6)	−0.0031 (5)	0.0101 (5)	−0.0043 (4)
C10A	0.0368 (6)	0.0237 (5)	0.0414 (7)	0.0052 (4)	0.0120 (5)	0.0000 (4)
C11A	0.0320 (6)	0.0298 (5)	0.0362 (6)	−0.0049 (4)	0.0105 (5)	0.0007 (4)
C12A	0.0351 (6)	0.0289 (5)	0.0250 (6)	0.0017 (4)	0.0075 (5)	0.0077 (4)
C13A	0.0491 (7)	0.0382 (6)	0.0252 (6)	0.0048 (5)	0.0107 (5)	0.0093 (5)
C14A	0.0422 (7)	0.0395 (6)	0.0353 (6)	−0.0043 (5)	0.0141 (5)	0.0136 (5)
C15A	0.0484 (7)	0.0333 (6)	0.0345 (6)	0.0091 (5)	0.0054 (6)	0.0118 (5)
O1B	0.0419 (4)	0.0208 (3)	0.0293 (4)	0.0014 (3)	0.0179 (3)	0.0031 (3)
O2B	0.0417 (4)	0.0246 (3)	0.0253 (4)	0.0046 (3)	0.0177 (3)	0.0045 (3)
O3B	0.0614 (5)	0.0276 (4)	0.0314 (4)	0.0090 (3)	0.0219 (4)	0.0026 (3)
C1B	0.0244 (5)	0.0206 (4)	0.0237 (5)	0.0012 (4)	0.0058 (4)	0.0041 (4)
C2B	0.0243 (5)	0.0258 (5)	0.0190 (5)	0.0016 (4)	0.0071 (4)	0.0051 (4)
C3B	0.0247 (5)	0.0235 (5)	0.0245 (5)	0.0042 (4)	0.0045 (4)	0.0054 (4)
C4B	0.0201 (5)	0.0261 (5)	0.0254 (5)	0.0001 (4)	0.0036 (4)	0.0072 (4)
C5B	0.0208 (5)	0.0309 (5)	0.0235 (5)	0.0007 (4)	0.0067 (4)	0.0078 (4)
C6B	0.0195 (5)	0.0286 (5)	0.0202 (5)	0.0014 (4)	0.0046 (4)	0.0012 (4)
C7B	0.0471 (7)	0.0253 (5)	0.0318 (6)	0.0068 (5)	0.0135 (5)	0.0054 (4)
C8B	0.0290 (5)	0.0257 (5)	0.0361 (6)	−0.0009 (4)	0.0069 (5)	0.0114 (4)
C9B	0.0423 (7)	0.0293 (6)	0.0591 (9)	−0.0062 (5)	−0.0059 (7)	0.0095 (6)
C10B	0.0364 (6)	0.0305 (5)	0.0613 (8)	0.0034 (5)	0.0112 (6)	0.0217 (5)
C11B	0.0562 (8)	0.0345 (6)	0.0534 (8)	−0.0021 (5)	0.0235 (6)	0.0196 (5)
C12B	0.0287 (5)	0.0322 (5)	0.0250 (5)	0.0032 (4)	0.0122 (4)	−0.0003 (4)
C13B	0.0427 (6)	0.0439 (6)	0.0335 (6)	0.0085 (5)	0.0210 (5)	0.0043 (5)
C14B	0.0378 (6)	0.0408 (6)	0.0322 (6)	−0.0012 (5)	0.0112 (5)	−0.0107 (5)
C15B	0.0373 (6)	0.0364 (6)	0.0377 (6)	0.0109 (5)	0.0159 (5)	0.0013 (5)

### Geometric parameters (Å, º)

**Table:**

O1A—C1A	1.3623 (11)	O1B—C1B	1.3645 (12)
O1A—H1A	0.885 (15)	O1B—H1B	0.883 (17)
O2A—C2A	1.3535 (12)	O2B—C2B	1.3567 (13)
O2A—H2A	1.154 (14)	O2B—H2B	0.974 (17)
O3A—C7A	1.2373 (13)	O3B—C7B	1.2436 (15)
C1A—C6A	1.3849 (15)	C1B—C6B	1.3899 (15)
C1A—C2A	1.4030 (14)	C1B—C2B	1.4011 (15)
C2A—C3A	1.4112 (13)	C2B—C3B	1.4078 (14)
C3A—C4A	1.4260 (14)	C3B—C4B	1.4269 (15)
C3A—C7A	1.4545 (15)	C3B—C7B	1.4450 (16)
C4A—C5A	1.3893 (14)	C4B—C5B	1.3830 (15)
C4A—C8A	1.5483 (13)	C4B—C8B	1.5482 (14)
C5A—C6A	1.4086 (13)	C5B—C6B	1.4124 (14)
C5A—H5AA	0.9500	C5B—H5BA	0.9500
C6A—C12A	1.5366 (15)	C6B—C12B	1.5380 (15)
C7A—H7A	1.001 (11)	C7B—H7B	0.981 (14)
C8A—C9A	1.5345 (16)	C8B—C10B	1.5284 (17)
C8A—C10A	1.5349 (16)	C8B—C11B	1.5350 (19)
C8A—C11A	1.5454 (16)	C8B—C9B	1.5355 (16)
C9A—H9AA	0.9800	C9B—H9BA	0.9800
C9A—H9AB	0.9800	C9B—H9BB	0.9800
C9A—H9AC	0.9800	C9B—H9BC	0.9800
C10A—H10A	0.9800	C10B—H10D	0.9800
C10A—H10B	0.9800	C10B—H10E	0.9800
C10A—H10C	0.9800	C10B—H10F	0.9800
C11A—H11A	0.9800	C11B—H11D	0.9800
C11A—H11B	0.9800	C11B—H11E	0.9800
C11A—H11C	0.9800	C11B—H11F	0.9800
C12A—C13A	1.5323 (16)	C12B—C13B	1.5335 (17)
C12A—C15A	1.5372 (17)	C12B—C15B	1.5347 (16)
C12A—C14A	1.5414 (16)	C12B—C14B	1.5382 (15)
C13A—H13A	0.9800	C13B—H13D	0.9800
C13A—H13B	0.9800	C13B—H13E	0.9800
C13A—H13C	0.9800	C13B—H13F	0.9800
C14A—H14A	0.9800	C14B—H14D	0.9800
C14A—H14B	0.9800	C14B—H14E	0.9800
C14A—H14C	0.9800	C14B—H14F	0.9800
C15A—H15A	0.9800	C15B—H15D	0.9800
C15A—H15B	0.9800	C15B—H15E	0.9800
C15A—H15C	0.9800	C15B—H15F	0.9800
			
C1A—O1A—H1A	110.9 (9)	C1B—O1B—H1B	112.1 (10)
C2A—O2A—H2A	107.9 (7)	C2B—O2B—H2B	105.0 (10)
O1A—C1A—C6A	121.43 (9)	O1B—C1B—C6B	121.39 (9)
O1A—C1A—C2A	118.85 (9)	O1B—C1B—C2B	119.02 (10)
C6A—C1A—C2A	119.71 (9)	C6B—C1B—C2B	119.58 (9)
O2A—C2A—C1A	115.16 (8)	O2B—C2B—C1B	115.32 (9)
O2A—C2A—C3A	122.78 (9)	O2B—C2B—C3B	122.63 (9)
C1A—C2A—C3A	122.06 (9)	C1B—C2B—C3B	122.02 (10)
C2A—C3A—C4A	118.86 (9)	C2B—C3B—C4B	119.22 (9)
C2A—C3A—C7A	116.16 (9)	C2B—C3B—C7B	116.57 (10)
C4A—C3A—C7A	124.98 (9)	C4B—C3B—C7B	124.17 (9)
C5A—C4A—C3A	116.85 (9)	C5B—C4B—C3B	116.59 (9)
C5A—C4A—C8A	119.49 (9)	C5B—C4B—C8B	119.99 (10)
C3A—C4A—C8A	123.65 (9)	C3B—C4B—C8B	123.41 (9)
C4A—C5A—C6A	124.74 (9)	C4B—C5B—C6B	124.97 (10)
C4A—C5A—H5AA	117.6	C4B—C5B—H5BA	117.5
C6A—C5A—H5AA	117.6	C6B—C5B—H5BA	117.5
C1A—C6A—C5A	117.68 (9)	C1B—C6B—C5B	117.45 (9)
C1A—C6A—C12A	120.97 (9)	C1B—C6B—C12B	120.95 (9)
C5A—C6A—C12A	121.34 (9)	C5B—C6B—C12B	121.59 (10)
O3A—C7A—C3A	124.01 (9)	O3B—C7B—C3B	124.19 (10)
O3A—C7A—H7A	115.1 (7)	O3B—C7B—H7B	117.6 (9)
C3A—C7A—H7A	120.8 (7)	C3B—C7B—H7B	118.2 (9)
C9A—C8A—C10A	106.64 (9)	C10B—C8B—C11B	105.50 (10)
C9A—C8A—C11A	106.03 (9)	C10B—C8B—C9B	111.32 (10)
C10A—C8A—C11A	110.55 (9)	C11B—C8B—C9B	106.67 (10)
C9A—C8A—C4A	111.89 (9)	C10B—C8B—C4B	111.07 (9)
C10A—C8A—C4A	111.07 (8)	C11B—C8B—C4B	111.32 (9)
C11A—C8A—C4A	110.48 (9)	C9B—C8B—C4B	110.77 (9)
C8A—C9A—H9AA	109.5	C8B—C9B—H9BA	109.5
C8A—C9A—H9AB	109.5	C8B—C9B—H9BB	109.5
H9AA—C9A—H9AB	109.5	H9BA—C9B—H9BB	109.5
C8A—C9A—H9AC	109.5	C8B—C9B—H9BC	109.5
H9AA—C9A—H9AC	109.5	H9BA—C9B—H9BC	109.5
H9AB—C9A—H9AC	109.5	H9BB—C9B—H9BC	109.5
C8A—C10A—H10A	109.5	C8B—C10B—H10D	109.5
C8A—C10A—H10B	109.5	C8B—C10B—H10E	109.5
H10A—C10A—H10B	109.5	H10D—C10B—H10E	109.5
C8A—C10A—H10C	109.5	C8B—C10B—H10F	109.5
H10A—C10A—H10C	109.5	H10D—C10B—H10F	109.5
H10B—C10A—H10C	109.5	H10E—C10B—H10F	109.5
C8A—C11A—H11A	109.5	C8B—C11B—H11D	109.5
C8A—C11A—H11B	109.5	C8B—C11B—H11E	109.5
H11A—C11A—H11B	109.5	H11D—C11B—H11E	109.5
C8A—C11A—H11C	109.5	C8B—C11B—H11F	109.5
H11A—C11A—H11C	109.5	H11D—C11B—H11F	109.5
H11B—C11A—H11C	109.5	H11E—C11B—H11F	109.5
C13A—C12A—C6A	112.27 (9)	C13B—C12B—C15B	107.54 (10)
C13A—C12A—C15A	107.87 (9)	C13B—C12B—C6B	112.18 (9)
C6A—C12A—C15A	110.06 (10)	C15B—C12B—C6B	109.49 (9)
C13A—C12A—C14A	107.79 (10)	C13B—C12B—C14B	108.16 (9)
C6A—C12A—C14A	108.50 (9)	C15B—C12B—C14B	110.12 (9)
C15A—C12A—C14A	110.33 (9)	C6B—C12B—C14B	109.32 (9)
C12A—C13A—H13A	109.5	C12B—C13B—H13D	109.5
C12A—C13A—H13B	109.5	C12B—C13B—H13E	109.5
H13A—C13A—H13B	109.5	H13D—C13B—H13E	109.5
C12A—C13A—H13C	109.5	C12B—C13B—H13F	109.5
H13A—C13A—H13C	109.5	H13D—C13B—H13F	109.5
H13B—C13A—H13C	109.5	H13E—C13B—H13F	109.5
C12A—C14A—H14A	109.5	C12B—C14B—H14D	109.5
C12A—C14A—H14B	109.5	C12B—C14B—H14E	109.5
H14A—C14A—H14B	109.5	H14D—C14B—H14E	109.5
C12A—C14A—H14C	109.5	C12B—C14B—H14F	109.5
H14A—C14A—H14C	109.5	H14D—C14B—H14F	109.5
H14B—C14A—H14C	109.5	H14E—C14B—H14F	109.5
C12A—C15A—H15A	109.5	C12B—C15B—H15D	109.5
C12A—C15A—H15B	109.5	C12B—C15B—H15E	109.5
H15A—C15A—H15B	109.5	H15D—C15B—H15E	109.5
C12A—C15A—H15C	109.5	C12B—C15B—H15F	109.5
H15A—C15A—H15C	109.5	H15D—C15B—H15F	109.5
H15B—C15A—H15C	109.5	H15E—C15B—H15F	109.5
			
O1A—C1A—C2A—O2A	−0.23 (14)	O1B—C1B—C2B—O2B	2.92 (13)
C6A—C1A—C2A—O2A	−179.75 (9)	C6B—C1B—C2B—O2B	−175.97 (8)
O1A—C1A—C2A—C3A	179.77 (9)	O1B—C1B—C2B—C3B	−179.19 (9)
C6A—C1A—C2A—C3A	0.25 (16)	C6B—C1B—C2B—C3B	1.93 (15)
O2A—C2A—C3A—C4A	−177.43 (9)	O2B—C2B—C3B—C4B	179.78 (9)
C1A—C2A—C3A—C4A	2.57 (15)	C1B—C2B—C3B—C4B	2.04 (15)
O2A—C2A—C3A—C7A	3.57 (14)	O2B—C2B—C3B—C7B	2.22 (14)
C1A—C2A—C3A—C7A	−176.43 (9)	C1B—C2B—C3B—C7B	−175.52 (10)
C2A—C3A—C4A—C5A	−2.79 (14)	C2B—C3B—C4B—C5B	−3.52 (14)
C7A—C3A—C4A—C5A	176.12 (10)	C7B—C3B—C4B—C5B	173.84 (10)
C2A—C3A—C4A—C8A	176.94 (9)	C2B—C3B—C4B—C8B	175.97 (9)
C7A—C3A—C4A—C8A	−4.15 (16)	C7B—C3B—C4B—C8B	−6.67 (16)
C3A—C4A—C5A—C6A	0.36 (15)	C3B—C4B—C5B—C6B	1.28 (15)
C8A—C4A—C5A—C6A	−179.38 (9)	C8B—C4B—C5B—C6B	−178.23 (9)
O1A—C1A—C6A—C5A	177.85 (9)	O1B—C1B—C6B—C5B	177.04 (9)
C2A—C1A—C6A—C5A	−2.65 (15)	C2B—C1B—C6B—C5B	−4.10 (14)
O1A—C1A—C6A—C12A	−3.19 (15)	O1B—C1B—C6B—C12B	−4.00 (14)
C2A—C1A—C6A—C12A	176.32 (9)	C2B—C1B—C6B—C12B	174.86 (9)
C4A—C5A—C6A—C1A	2.40 (16)	C4B—C5B—C6B—C1B	2.58 (15)
C4A—C5A—C6A—C12A	−176.56 (10)	C4B—C5B—C6B—C12B	−176.38 (9)
C2A—C3A—C7A—O3A	−2.33 (16)	C2B—C3B—C7B—O3B	−1.77 (17)
C4A—C3A—C7A—O3A	178.74 (10)	C4B—C3B—C7B—O3B	−179.20 (10)
C5A—C4A—C8A—C9A	3.81 (14)	C5B—C4B—C8B—C10B	123.69 (11)
C3A—C4A—C8A—C9A	−175.91 (10)	C3B—C4B—C8B—C10B	−55.79 (13)
C5A—C4A—C8A—C10A	−115.24 (11)	C5B—C4B—C8B—C11B	6.45 (14)
C3A—C4A—C8A—C10A	65.03 (13)	C3B—C4B—C8B—C11B	−173.02 (10)
C5A—C4A—C8A—C11A	121.69 (11)	C5B—C4B—C8B—C9B	−112.06 (12)
C3A—C4A—C8A—C11A	−58.03 (13)	C3B—C4B—C8B—C9B	68.47 (14)
C1A—C6A—C12A—C13A	−178.10 (10)	C1B—C6B—C12B—C13B	−176.58 (9)
C5A—C6A—C12A—C13A	0.83 (15)	C5B—C6B—C12B—C13B	2.35 (13)
C1A—C6A—C12A—C15A	61.73 (12)	C1B—C6B—C12B—C15B	−57.26 (12)
C5A—C6A—C12A—C15A	−119.34 (11)	C5B—C6B—C12B—C15B	121.66 (10)
C1A—C6A—C12A—C14A	−59.08 (14)	C1B—C6B—C12B—C14B	63.45 (12)
C5A—C6A—C12A—C14A	119.85 (11)	C5B—C6B—C12B—C14B	−117.63 (11)

### Hydrogen-bond geometry (Å, º)

**Table:**

*D*—H···*A*	*D*—H	H···*A*	*D*···*A*	*D*—H···*A*
O1*A*—H1*A*···O2*A*	0.885 (15)	2.169 (15)	2.6360 (10)	112.4 (11)
O2*A*—H2*A*···O3*A*	1.154 (14)	1.484 (14)	2.5013 (10)	142.7 (12)
O1*B*—H1*B*···O2*B*	0.883 (17)	2.212 (16)	2.6443 (10)	109.7 (12)
O2*B*—H2*B*···O3*B*	0.974 (17)	1.608 (16)	2.5046 (10)	150.9 (16)
O1*B*—H1*B*···O3*A*	0.883 (17)	1.916 (17)	2.7485 (11)	156.4 (15)
O1*A*—H1*A*···O3*B*^i^	0.885 (15)	1.912 (15)	2.7289 (11)	152.9 (13)

Symmetry code: (i) *x*, *y*−1, *z*.
